# Bilious

**Published:** 1875-08

**Authors:** 


### BILIOUS.

This is a most convenient and comprehensive word. It is ever ready to come to the aid of the doctor when a too inquisitive patient asks troublesome and foolish questions. If told that he is "bilious," that is enough to satisfy him of the nature of his disease and the diagnostic skill of his physician. Very likely he thought so himself, and if the doctor is of the same opinion the question is settled. Moreover, it is quite probable that both are right. If the liver fails to secrete the proper quantity of bile, the process of digestion is imperfectly performed. If the bowels are constipated and the discharges are of a light clay color, the patient is bilious. Should the secretive function of the liver cease entirely more serious symptoms would occur, and in time a fatal result.

If there is increased secretion, what is sometimes called an "overflow of bile," producing an attack of vomiting and diarrhœa, then the patient evidently has a "bilious turn." The re-absorption of bile after its formation in the liver, gives rise to the most obnoxious bilious symptoms. The coloring matter of the bile is easily detected in the urine. and the jaundiced tinge of the eyes and skin indicate that the disorder is clearly *bilious*.

And so we see the popular term applied, and quite appropriately too, to three or four morbid conditions. It is not enough for a physician to know that his patient is bilious. In order to prescribe intelligently he must understand which of the several biliary derangements he is required to remedy. Is the liver torpid, or is it stimulated to an unnatural activity? Or, does the third condition exist, in which the bile once secreted has been absorbed?

The folly of taking pills, bilious, or anti-bilious, or bitters that are good for the liver, or any medicine indiscriminately, requires no further demonstration.

There is one course which may be always adopted safely and advantageously as a preventive of bilious complaints in the hot weather of summer, and that is to live on a light and unstimulating diet. But little meat—and perhaps many persons would be, better if they abstained entirely—is the safest rule in summer. Eat fruit and vegetables, drink milk and water, and you will not be likely to suffer from biliousness.

But should disease come, with symptoms of biliousness, then commit your case to the management of a physician who will doubtless have the acumen to decide which of the different morbid conditions exists, and direct his treatment accordingly.

---

THE NEW PILE CURE.—Scarcely anything we has written of late has attracted as much attention as the little item on the application of dry cold, for the relief of piles or hemorrhoids. We have prescribed cold sitz-baths for years, with general success; but have found that intenser cold than can safely be applied to an extended surface, is usually necessary for permanent results. What we have sought, but never found till now, is the power to concentrate the cold at the inflamed and congested point. This we can do perfectly by the use of Dr. Mattson's siphon cone. We have prescribed it in cases where the patient could neither walk nor stand, nor sit nor sleep, so intense was his suffering, and have seen absolute freedom from all pain secured in the space of one minute. The theory of relief and cure is simple and philosophical, and we will cheerfully explain it if desired. Meantime, let us remark, that the druggists keep this very valuable appliance for sale; or if they do not, they certainly should.

---

One false step, one wrong habit, one corrupt companion, one loose principle, may wreck all your prospects, and all the hopes of those who love, honor, and regard you.

